# Evidence of the Spacing Effect and Influences on Perceptions of Learning and Science Curricula

**DOI:** 10.7759/cureus.21201

**Published:** 2022-01-13

**Authors:** Xuechen Yuan

**Affiliations:** 1 Department of Education, Lakehead University, Thunder Bay, CAN

**Keywords:** massed learning, judgment of learning, memory, science education, spacing effect

## Abstract

The conventional science curricula generally favour educational practices that yield high scores on immediate examination, though it may not accurately predict students’ long-term academic achievement. In view of the pre-exam cramming phenomenon, this article shows the evidence of spacing effect in science education and probes into its theoretical mechanisms, effectiveness in experimental settings, and current applications in science learning. In brief, spacing works by repeatedly presenting the learning material across various temporal intervals. This paper suggests that spacing could significantly result in greater memory strength by alleviating multiple neurocognitive and behavioural properties of learning that are hampered by cramming. Together with the analysis of its relevance in science education, the spacing effect may further provide leverages for promoting long-term conceptual understanding and reflective skill development. However, there are many reasons that students and teachers may not be aware of or fully appreciate its benefits. Finally, this article discusses systemic barriers to why spaced repetition is underutilized in science curricula.

## Introduction and background

Common sense seems to dictate that school grades reflect students’ knowledge and understanding of tested subjects [1]. While grading allows educators to provide a baseline evaluation for learners’ progress, it does not necessarily follow that such assessment is fair for every student. The curriculum based on positivist epistemology is a product of Eurocentric narratives, whose main purpose is to produce standardized knowledge [2]. Critiques have it that conventional curricula tend to omit the problems that lead to the reproduction of the dominant educational methods, thereby marginalizing other useful pedagogical approaches [2]. A common phenomenon in the conventional classroom is massed learning in which students intensively study things before exams [3]. Plainly put, students study a sheer volume of information at once to obtain immediate recall or recognition on the test items, which is dominant in practices among university students majoring in science subjects [1]. Kelley and Whatson suggest that the underlying assumption of such behaviour concerns both individual learning patterns and current teaching standards, since the outcome of conventional science education often comes into play with students’ heavy workload and resistance from school inspectors to challenge its efficiency [4].

For decades, a great deal of attention has been directed to the principle of spacing effect (or spaced repetition/spaced practice) [5]. The spacing effect was first discovered by German philosopher Ebbinghaus with a humble approach to studying memory. By simply creating a study list on pieces of paper, Ebbinghaus found that he could master his memory of items faster if the repetition of items was spaced out at different intervals [6]. This phenomenon has real-life applications. For example, television commercials separated by a number of non-repeated commercials are more likely to be remembered [6].

**Figure 1:**
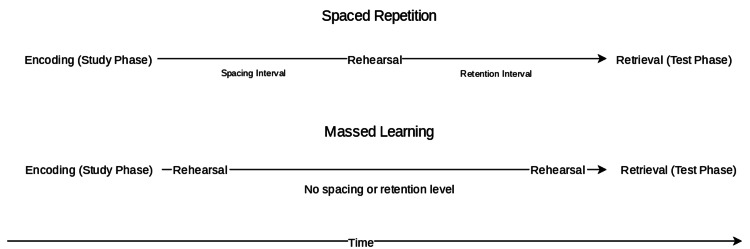
A basic illustration of spaced and massed practice in instruction. Note that time during each interval may not be identical.

In a general sense, spaced repetition is an evidence-based information encoding technique that improves recall efficiency by dividing the enormous content into a series of short-piece information across temporally spaced intervals (see Figure 1). Cepeda et al. demonstrated in a meta-analysis that, in the case of equal total study time, recall efficiency was higher in spacing with expanding inter-study interval than fixed interval and massed learning [7]. Kornmeier and Sosic-Vasic also show that science learning sessions with spaced repetition could double the efficiency of massed instruction [8]. Spaced repetition takes various forms that show spacing effects, such as interleaving and micro-learning [9,10]. As a result, under the spacing principle, various educational software applications have been developed to help optimize learning.

In psychological research, spaced repetition opens up new possibilities in the area of psychological sciences to explore, replicate, and summarize the efficiency and potential for spaced practice in education, including second language instruction and science education [11,12]. A large body of research has described the universal benefit of spaced repetition on the long-term retention of content across disciplines, learning contexts, and geopolitical circumstances, providing compelling evidence for its validity. Despite breakthroughs, spaced repetition often struggles to penetrate its benefit into traditional curricula [4,5].

This paper aims to focus exclusively on the impact of spaced repetition on science education, due in part to the dubious reputation of using standardized tests in science courses to evaluate students’ mastery [12], and partially it enables researchers to bridge the research gap that explores the link between memory retention and reflective skills that make up two fundamental objectives for scientific learning [13]. The traditional way to measure the spacing effect is to compare memory performance following immediate and delayed reinforcements, but the moderating role of reflective skills on spacing is overlooked [1,14]. This gap must be addressed because, first, spaced repetition is a research interest in memory science but not in other disciplines of psychological science [15]. Second, the development of reflective skills is now widely regarded as one of the fundamental objectives of science instruction after decades of metacognition research [13]. Despite this fact, only a limited number of studies have provided direct evidence of the benefit of reflective skill development in science education and in obtaining the spacing effect [14,15]. Therefore, this paper aims to provide a more comprehensive understanding of the spaced repetition principle from the reflective and social dimensions of learning by integrating existing research. It is aimed at lay audiences and does not require expert knowledge of the field. The paper will examine the direct mechanism of human memory and information processing underlying the spaced effect, as well as indirect effects of self-regulation of knowledge, a cognitive bias in learning, and school expectation of learning. However, the paper does not expect to address specific issues related to fixed-versus-varied repetition timing, algorithms, teacher education, and policymaking for spaced practice. Most importantly, the paper’s purpose is to look at the evidence of spacing effect in science education, and the potential to implement spaced repetition in educational practice. The paper will address two questions:

(1) What is the evidence, socially and cognitively, that spaced repetition improves learning and test results for science subjects?

(2) What are the potential and barriers to utilizing spacing effect in science education, socially and cognitively?

The impact of the spacing effect on memory and learning outcomes is predicated on complex neural and cognitive mechanisms. Understanding the interplay of spaced practices and these mechanisms can shed insight into the utility of spacing in science curriculums, and critiques on conventional instructional practices [1]. This paper explores four mechanisms/themes of learning integral to understanding the spacing effect: biological, cognitive, metacognitive, and social. Those mechanisms have some interconnected elements and the relative influences of each are reported in this paper.

## Review

Biological foundation of learning

Synaptic Plasticity and Spacing Effect

The biological mechanisms of learning and memory are operated by modifying the connection strength between central nervous system neurons. Synapse is the site where two neurons join together through the transmission of nerve impulses and the release of neurotransmitters. Memory consolidation is a procedure in which neurons are created, linked, and (re)activated with their various patterns and firing rates. Repetition of specific memories can strengthen the existing synaptic connections and produce more synapses for long-term encoding, whereas unused memory gradually promotes weakening and apoptosis of synapses. This ability is called synaptic plasticity [10].

While repetition can aid in memory recall by reinforcing synaptic connections, it does not necessarily expound on whether this effect is long-lasting and could face up to the offshoot of mental fatigue. Learning, like many neural activities, requires a refractory period that separates two successive stimuli [16]. For example, massed instruction - depriving refractory period between repetition trials - may increase the risk of tiredness that could trigger “serious cognitive decline in individual performance” and promote rapid decay of temporary memory [4,10].

At the neuronal level of learning, synaptic transmission is facilitated by the production, uptake, release, and disposal of excitatory and inhibitory neurotransmitters, which activate memory traces in response to stimuli (e.g., study items). For example, excitation of serotonin [17], glutamate [18], dopamine [19], gamma-aminobutyric acid (GABA) [20], and acetylcholine [21] receptors in the hippocampus is vital for regulating learning, concentration, and memory expression. Next, these neurotransmitters must be continuously rebuilt to fuel the signal transduction and prolong the cascade in memory consolidation. Mental fatigue indicates that these chemical reactions are inhibited when presynaptic neurons are not able to reassemble and release neurotransmitters consecutively for cognitive activities, due to over-stimulation caused by massed learning. Therefore, spacing out learning sessions can sufficiently avoid mental fatigue by overcoming the refractory period by regulating the synthesis of neurotransmitters and signalling nerve impulses at a steady pace, which could successively aid in the long-term storage of memory traces [10].

The impact of spaced repetition on learning rigorously follows synaptic plasticity mechanisms. Kelley and Whatson and Smolen et al. suggest that progressive repetition of stimuli separated by irregular spacing intervals can lead to a long-lasting enhancement of synaptic contact signalling - long-term potentiation (LTP) [4,16]. Theoretically, LTP leads to the formation of long-term memory (LTM) via multiple sequences. First, initial learning will generate short-term memory (STM) traces by transmitting nerve impulses to the neighbouring synapses. These impulses create a cascade reaction in the postsynaptic neurons, thus aiding in the transfer of STM into long-term storage [8]. LTP induction is a dynamic process; that is, signalling cascades during LTP installation can exhibit very distinctive intracellular activity patterns (e.g., protein synthesis and kinase activity) influenced by temporal factors such as the timing of spacing intervals [8]. Thus, learning consolidation tends to be the strongest when the scheduling of spacing concurs with peak intracellular activities. That being said, it is possible to “[make] LTM in minutes” with dynamic and optimal spaced schedules [4].

Cortical Response to Spaced Learning

The spacing effect delineates a good representation of brain areas associated with memory formation. Kornmeier and Sosic-Vasic epitomize that learning and memory exist in “different areas of the brain, like the amygdala, hippocampus, cerebellum, and cortex” [8]. This opens up potential research opportunities to investigate the impact of spaced schedules on cognitive and emotional skills and motor memories.

By using EEG in memory research, researchers can observe the brain voltage potential in response to task-specific activities. N400 is an EEG waveform component that reveals the LTM retrieval strength of lexical items [22]. Similarly, the late positive component (LPC) is responsible for the general recollection process [18]. In the massed learning session, Zhao et al. discovered both a reduced response of LPC and significant suppression within the N400 component, accordingly indicating that the recollection process and long-term recall ability of memory traces were impeded [23]. In contrast, spaced repetition led to greater memory performance and rising LPC signals that implicated a stronger representation of prior learning and recollection of previously learnt items [23]. Overall, it is evident that the biological mechanism of the spacing effect can be adopted by potential pedagogical practices and technologies to consolidate learning.

Cognition

Spacing Effect on Long-Term Memory

The standard way of thinking about cognition holds that it involves complex mental processes of obtaining, understanding, retaining, and retrieving information. One of the most important information processing characteristics in cognition is memory. Learning begins with creating temporary memories that are stored in STM. Through repeated encoding and rehearsal, these specific memories can be transferred to LTM storage, making them last indefinitely [4,10].

Encoding study materials distributed throughout the spaced trials facilitates the transfer of materials to LTM storage, which involves the serial position effect. The underlying mechanism of the serial position effect is that the first and last component in a study list will have a more robust mental representation of memory. Specifically, the primacy effect reveals that the earlier items in a study list are more likely to be converted into LTM, just as how important the first impression is in a long-term social interaction [10].

The primacy effect can lead to a rapid accumulation of LTM with spaced learning. Kelley and Whatson suggest that memory performance after just an hour of spaced repetition can hold up to four months of massed instruction, showing a high level of LTM encoding [4]. Theoretically, the primacy effect underlying the spaced repetition works by grouping together the initial study items from short spaced sessions into a bigger memory unit (chunk) that is subconsciously rehearsed during spacing intervals [10]. Accordingly, the large study list can be reduced to a few chunks. Spaced learning can protect earlier chunks of memories from the interference of incoming stimuli (retroactive interference), but massed learning would trigger rapid degradation and elimination of earlier memories because the chunk cannot be formed readily when massing [24].

Spacing Effect on Short-Term Memory

The capacity of LTM to store prior information is infinite; STM, on the other hand, stores recent memory traces with little or no consolidation and can disappear spontaneously [25]. The transition of STM to LTM usually relies on memory rehearsal involving recall and general recollection to prevent the inevitable decay of immediate memory traces and interference from rivalry stimuli [25]. Besides the primacy effect, another representation of the serial position, the recency effect, describes the tendency to remember recently occurring details. This cognitive bias may unintentionally impact students’ preference for their study schedules and loads.

Shail explains that “in the recency effect, items are stored as short-term… declines with the passage of time and by the presentation of additional information” [4]. This argument suggests that memory traces stored in STM are susceptible to retrospective interference if they are not repeatedly reinforced. In any case, students may opt for massed learning over spaced repetition for a number of reasons. First, the registration of memory traces in STM from massed instruction provides a more salient representation for immediate conscious recollection and mental operation, thereby reducing effort for momentary memory retrieval [26]. Additionally, massed learning may lead to stronger temporary retrieval strength that is useful for immediate testing. It also shows a significant occurrence of repetition priming - a rapid enhancement in responding to similar stimuli due to the residual effect of massed conditioning, which may ultimately help students to respond more spontaneously to directly tested and associated test items [23].

Contrary to momentary benefits of massed learning in STM retrieval capacity, Zhao et al. used EEG to show that LTM retrieval of lexical knowledge might be hampered under the massed teaching condition by observing reduced activities of N400 and LPC waveform signals [23]. Using a similar method, Kim et al. and Van Strien et al. identified a higher peak amplitude of N400 in spacing than the massed condition with no sign of attenuation; LPC signal, by contrast, showed larger peak responses but with smaller peak latencies in massed repetition, which means better and faster recognition of study items on the immediate testing with less effortful memory processing [27,28]. This shows a large effect of repetition priming. However, repetition priming may deteriorate LTM encoding for study items due to less conscious/semantic processing of previously encountered items [28]. Taken together, these findings suggest that educators and students may find cramming beneficial for immediate memory assessment but overlook its potential harm to long-term retention.

Spacing and Motivation

Motivation plays a crucial role in facilitating LTM retention. It may derive from external incentives (i.e., reward and punishment) or/and intrinsic incentives (i.e., personal curiosity) [29]. During the learning process, students, driven by external incentives such as high scores in exams, are more likely to focus only on unfamiliar items to “recognize” them in exams, whereas items “recognized” but not yet “rehearsed” are skimmed. As a result, these items may never be recalled outside of testing, resulting in limited access to LTM storage [23,29]. On the contrary, students with high intrinsic motivation might find studying more effortful and thus develop personal insights into the content. Therefore, spaced repetition may indirectly influence LTM storage strength through the intermediary of intrinsic motivation. Specifically, it elevates students’ learning capacity, self-efficacy, and incentive for self-growth, while controlling for their baseline proficiency [29]. The underlying reason is that spaced repetition helps students maintain the conscious effort to continue engaging with the spaced learning schedule.

Spacing and Attention

In addition to the impact on memory, it is also predictable that motivation might help direct attention, leading to less mind wandering (i.e., zoning out). Mind-wandering may occur frequently in lengthy and difficult sessions due to spontaneous distractions that govern STM capacity [30]. However, appropriate educational support, including pedagogical resources and repetition schedules, may mitigate this impact. Under massed instructions, students tend to undermine the potential utility of such resources. Specifically, mind wandering emerges when students begin to cease internal mechanisms of inhibition control and stop receiving additional information over a long learning session without breaks. It may also occur when students perceive immediate items in the massed session to be either too redundant or too arduous [30]. Contrariwise, Metcalfe and Xu found that students focused on learning more in the spaced practice, pointing to the complex attention processing mechanisms influenced by internal attributes such as “level of processing” and external variables involving task difficulties and rehearsal schedules [30].

Overall, it is evident that while spaced repetition may not show immediate benefits on classroom examinations, its effects on learners’ long-term retention, motivation, and attention are long-lasting. It also implies that current educational practices and policy initiatives are often short-sighted and profit-oriented, undermining incremental progress for learners.

Metacognition

Judgements of Spaced Learning

Learning involves reflective components that regulate the basic information processing in cognition, helping learners inspect their learning progress and respond to challenges. This skill is often known as metacognition [31]. Conventional wisdom has it that metacognition focuses on higher-order thinking - thinking beyond cognition, which is extremely important as students are expected to have introspective skills that go beyond factual learning [32].

Metacognition can be assessed by judgements of learning (JOL), which investigates people’s self-awareness on how well they remember or understand the given materials. Typically, in the cued-recall test consisting of multiple word pairs (e.g., chicken-cow), making JOLs after the presentation of each word pair facilitates “the associative relationship” and meaning-making “between the cue and target”, leading to improved recall performance of STM and LTM compared to many who did not [33]. In particular, the effort of generating JOLs is fruitful for encoding word pairs whose intrinsic meanings are relative to participants [33]. In addition to its benefits, the current study examined whether students could accurately describe the advantages of spaced repetition through JOLs.

Cohen et al. and Logan et al. suggest that students’ decision to make JOLs under two repetition conditions depend on cognitive bias and complex issues [34,35]. Their studies found that, whether or not students might be unaware of its benefits, they showed a clear preference in JOLs for massed repetition over spaced repetition. Students' preferences may be intuitive, that spaced items may be more detached from STM and not readily available for immediate retrieval [34,35]. The reliance on temporary fluency and accessibility is an important factor in the creation of JOLs. In other words, students perceive that repeating items in massed trials yield a greater coherence with their initial presentation than spaced items. Moreover, students often do not know future memory (LTM) assessments when making JOLs, leading to the captivity of spaced repetition from a momentary gain perspective [35].

Since the memory encoding process during the initial presentation of items is weak, preference for massed learning may also contribute to the need to increase perceptual retrieval strength to avoid forgetting [35]. Cohen et al. found that students tended to choose spaced schedules for easier items while preferring massed practice toward rehearsal of more complicated items since memory traces of difficult items tend to fade away faster than that of more comfortable items [34]. For students who preferred spaced schedules, their objective might be simply to “minimize the temporal distance between the initial presentation of an item and when they are tested on it” [34].

Similarly, Kornell and Bjork conducted a study in which participants were asked to pair artwork with the artist’s name through recognition and recall tests, after studying artworks from a particular artist continuously (massed) or mixing them with others’ artworks (spaced) [26]. Although participants performed better on both recall and recognition tests when the specific artist’s artworks were spaced apart, they perceived massing as more efficient because of a sense of ease and proximity. Together, these studies shed insight into the long-lasting influence of conventional teaching methods favouring short-term cramming, at the expense of students’ long-term retention and judgements for successful learning strategies.

Affective Processing in Spaced Learning

As mentioned earlier, repetition priming (massed conditioning) tends to reduce the effort of LTM encoding since the initial representation of items may create ease of processing at their next occurrence [28]. Under this assumption, priming under the massed condition will lead to a shallower semantic/affective and more automatic processing than spaced practices. In succession, insufficient semantic/affective processing may also be fed back to attenuate the repetition priming and the spacing effect [36].

The association of these factors generates a negative feedback loop on the memory consolidation of study items, which has real-life implications. Students tend to take the study content for granted (priming) while focusing their attention only on its physical property, undermining its intrinsic affective/semantic quality, which in turn impairs both immediate behavioural feedback and extended response (spacing effect) to materials they once could easily process [37]. In response, the curricula could increase the affective quality of the study content and direct students to explore abstract components in both emotion-charged and neutral materials, which can promote a more significant spacing effect and subsequent memory performance [36]. Overall, the spacing effect highlights the role of affective connotation in enhancing meaningful learning and memory efficiency.

Spacing and Critical Thinking

Based on critical reflection about factual content through different lenses, students may be more equipped to judge how they would gain conscious control of these “lenses”. Currently, not much research has been conducted on the relationship between spacing and critical thinking. In a study conducted by Foot-Seymour et al., students were taught to provide a critical website evaluation within a few days (massed) or weeks (spaced), with a set of given guidelines [38]. Not surprisingly, during the learning phases, students who received mass instruction were initially more effective at memorizing, recalling procedures, and engaging in critical thinking discourse than the spaced group. However, they exhibited low retention of critical thinking in the testing period. In contrast, students under the spaced condition described more reasoning based on their evaluation guidelines [38]. This study, combined with previous results, suggests that the spacing effect is present in memory consolidation of both critical thinking and factual learning skills. Therefore, learning efficiency under spaced repetition could be improved through the holistic development in inner psychophysical characteristics, and the ability to analyse, modulate, and empathize with one’s cognitive pattern in response to external stimuli.

Educational relevance in science education

Effectiveness of Spacing in Science Education

The (meta)cognitive advantages associated with spaced repetition are demonstrated in the experimental context. However, more research is needed to expand the theoretical basis for applying spaced learning in complex educational settings, especially in science learning. This paper incorporates mathematics (problem-solving) and biology (conceptual learning) to represent science curricula.

First, Kelley and Whatson suggest that the application of spaced learning allows biology courses to deliver concepts at an extraordinary speed and accelerate learning efficiency without compromising students’ attention and cognitive capacity [4]. This is achieved by implementing multiple short condensed biology classes with intervals filled with distractor activities such as physical exercise - an ideal way to minimize mental process and retroactive interference during spacing intervals. Such course design can significantly improve students’ long-term retention of undergraduate biological concepts, realizing the learning potential that could occur at an exceptionally rapid pace [4].

Such curriculum design can improve learning efficiency during memory encoding of advanced concepts. Still, consolidation and maintenance of these memories in spaced repetition could be more time-consuming as students need to regularly revisit these difficult concepts. Kerfoot discovered a parallel relationship between study time and learning efficiency in biomedical education [39]. Compared to students who restudied the entire concept list (similar to massed learning), those who only repeated incorrect or unfamiliar items in each study schedule took longer to complete learning (possibly due to repeated rehearsals of items that had been correctly answered before but then answered incorrectly). However, this repetition caused them to show greater incremental knowledge acquisition across each study schedule. Similarly, Taveira-Gomes et al. also suggest that additional time on learning can improve the recall accuracy of biomedical knowledge [40].

In mathematics education, it is no surprise that the benefit of spaced learning is resistant to forgetting. Despite its many advantages, there grows up a controversial issue on whether massed mathematical practice must be replaced entirely by spaced repetition or not [1,5,41]. In opposition to biology with mostly conceptual memorization, the ability to solve mathematics problems is the leading determinant to mathematics success [42]. Rohrer shows that spacing of the practice problems (rather than study schedule) improves mathematics learning and alleviates mental fatigue [43]. But it is also more complex in the classroom. For example, creating massed assignments immediately after classes may be an ideal strategy to familiarize students with the procedural approach and data processing [9]. As shown here, pure spacing of practice problems may not be necessary for mathematics learning, which requires a certain degree of massing (e.g., massed review sessions and blocked assignments) to allow students to focus on problems of similar kinds [9].

Interleaved Practice

Based on the above suggestions, educators and students in mathematics education must be cautious not to rely exclusively on the overarching principle of spaced repetition, thereby disregarding alternative education approaches that might be more appropriate for specific subjects. Interleaved practice is a derivative principle of spaced repetition, a strategy for rearranging problem sets designed for one topic into multiple topics in each course material/assignment (e.g., ABC ABC ABC instead of AAA BBB CCC.) It juxtaposes “different kinds of problems in an interleaved order” for each assignment so that “spacing intervals between consecutive problems of the same kind might expand” [9]. Hence, such practice produces a spacing effect for question orders instead of the timing of repetition. Rohrer et al. indicate that spacing out similar types of problems into different assignments can promote a better test outcome than blocked practices, as it trains students’ capacity to solve problems by comprehensive strategies of their choices rather than by rote memorization and repetition priming of procedural steps [9].

Micro-Learning

Another derivative practice embedded in spaced repetition mechanisms is micro-learning, which operates by breaking a large chunk of information into smaller learning units with the knowledge required (e.g., A B C instead of AA BB CC) to prevent mental fatigue and memory interference [10]. Learners can readily appreciate the prevalence of micro-learning through the skyrocketing popularity of educational technology. The mobile and online learning platforms have become indispensable instruments being used by schools and self-paced instruction. There are a plethora of open online micro-courses (MOOCs) currently developed and partnered with the world’s most reputational institutions and non-profit education providers, such as “Khan Academy™, Udemy™, Coursera™… edX™” [10]. It will be arbitrary to assert that micro-learning could replace contemporary educational practice. However, it can undoubtedly enrich learners' enthusiasm for autonomous learning through the medium of mobile technology. Therefore, sufficient skill and behavioural training on technology-based learning for students and educators could facilitate fruitful learning outcomes and regulate the potential distraction induced by electronic devices [10].

Through curriculum design and mobile device assistance, a few derivative spaced practices inspired by the spacing effect have been demonstrated in the real pedagogical application, to strengthen memory storage of conceptual knowledge and comprehensive skill modelling. Additionally, due to the growing popularity of educational technology, it would be ideal for further research to take an open-source application and change the spaced timing structures based on psychological evidence or academic recommendations made by educator-researchers, then conduct experiments to evaluate its effectiveness. This would allow for both within- or between-subjects designs.

Barriers of Spaced Practices in Education

For researchers, the advantages of the spacing effect are becoming increasingly evident; however, many overlook its practical values in the curricula [1]. This phenomenon could be examined at both individual and systemic levels. At first, when the available period for review is short, students have a clear preference to opt for a repetition schedule proximal to initial learning or examination, indicating that they are more inclined to relearn materials for short-term retention and immediate examinations [29]. Other interpretations suggest that using massed repetition for memory encoding provides more ease and fluency for the responsiveness of immediate conscious retrieval [10,35]. However, doing such is not ideal for LTM enhancement.

The foundation for the cramming phenomenon is embedded in the curriculum design dominating conventional curricula, which illuminates the impact of massed practices on classroom issues such as the amount of homework, test frequency, teacher feedback, and level of mastery [5]. When determining course materials and how the class should be organized, educators’ perception of learning might be engendered by the status quo. To further elaborate, many teachers are more comfortable with the massed educational practice, in which class topics are arranged into separate sections, and may be followed by one spacing interval - a final review session covering all topics before the test [1].

Through the implementation of a review session, it can be seen that some educators hold a certain degree of understanding of the spacing effect, regardless of whether they may like it. However, lack of institutional support and resources hinders their desire to further integrate spaced practices in the pedagogy. Lindsey et al. argue that providing and planning for spaced practices in the classroom “is beyond what any teacher or student can reasonably arrange” [44]. In most cases, educators’ instructional strategies are consistent with course materials (e.g., textbooks and assignments) and are organized into various chapters [1]. Therefore, spacing can be difficult if the book chapters and practice problems on relative topics still appear in blocked orders. But if questions and lessons in the textbook are not changed with only question orders rearranged, educators may find spaced practice relatively at ease, which may increase the chance that they will utilize it [9].

Nowadays, the validity of textbooks with spaced repetition is still debated. For example, the learning objectives of Saxon texts, which primarily use interleaved practice, are criticized by many educators on their learning objectives, with one research report determining that Saxon texts do not certainly enhance test performance [43]. This is because Saxon’s efficiency is hindered by features other than spacing, such as that it introduces new concepts when students have not yet mastered old concepts, and its focus on the “mastery of procedures at the expense of conceptual understanding” [9,45]. This finding suggests that more research is needed to explore the possibility of restructuring the textbook by spacing out the chapters throughout the book (reducing each chapter’s length) and its impact on students’ memory formation, motivation, attention, and learning efficiency.

Future Application and Research of Spacing Effect in Science Curricula

Overall, even though the spacing effect reveals its important theoretical and practical values in learning, its utility in conventional education and teacher practices has not become evident. It would also be a waste of effort to completely disprove the functionality of massed practice and is against present research findings suggesting that some degree of massing is necessary for learning biology and mathematics. So this paper requests conventional science curricula to establish smart goals for the stepwise development of spaced practices. The first suggestion is for educators to revisit the content repeatedly over the duration of the course, so each consecutive repetition will increase the depth of knowledge and allow new perspectives to emerge. Next, the paper calls for the incorporation of multimodal educational resources in the curricula, be it digital tools such as mobile and computer devices with adaptive algorithms, MOOCs, websites/applications with customized “flashcards” features based on prior recall accuracy, and interleaved textbooks with rearranged assignment questions (more research is needed). Ultimately, these resources could be an excellent asset for spacing to operate in a friendly and cost-effective way, potentially reducing pressure on teachers and making spacing a more rewarding experience.

Future research is needed to discover and evaluate potential strategies to illustrate the spaced repetition principle in science learning. It is the work of curriculum design research to help prepare classrooms that appropriately reflect the utilization of the spacing effect. A simpler suggestion and easier-to-implement experimental design would be to structure a course module or modules of what we know about the spacing effect and compare student performance at the designated time with that of a standard blocked approach, which would take minimal effort and provide significant data and evidence.

Future research in spaced repetition also needs to generate a complete knowledge of the diverse student groups’ learning experiences. The research could proceed with an extensive qualitative analysis beyond the quantitative methodologies. A mixed-method design might be of particular interest. These approaches may provide a thorough contextualized investigation of the current spaced practices, accounting for the needs of students who require additional support based on their narratives. Also, mixed-method research would help create better metrics for assessing the qualities of spaced practices in science education.

Also, research of spacing effect was predominantly conducted by controlling for students’ baseline knowledge proficiency to differentiate the effectiveness of spaced and massed practice in learning. It rarely assesses individual capabilities for those with learning difficulties and challenges. Under the circumstances, a standardized spaced educational approach - preserving the same volume of course load and examinations for each student - might be less effective than scaffolding. More research needs to tackle spaced repetition in a personalized domain, such as adaptive spaced schedules.

Furthermore, research in spaced repetition should incorporate multiple behavioural and sociopolitical determinants to its efficacy on learning and memory. Intervening variables such as post-learning mental activities, sleep functions and circadian rhythms, and use of technology could be examined as the independent variables in examining spaced learning outcomes, as retention intervals loaded with intensive cognitive activities will significantly increase the likelihood of forgetting. It is also of utmost importance to connect research findings on spaced learning to education policymaking. Yet, it might require a macro-level and constructivist interpretation of societies and ideologies.

## Conclusions

Spaced repetition is a fruitful tool for facilitating conceptual and reflective learning skills. Yet, its benefit is mainly exemplified in experimental settings and self-regulated learning. The paper demonstrates that the spacing effect can significantly improve learning, but not the judgement of learning. Together, the paper highlights some important insights. First, the spacing effect promotes LTM consolidation. Next, spaced practices result in a more robust enhancement of LTM encoding, recall, and recollection through both direct and indirect effects of memory processing characteristics. Contrastingly, massed practices increase instantaneous (STM) retrieval strength but reduce LTM encoding. Additionally, there are clear advantages to using spaced instruction in science curricula. However, as the cramming phenomenon becomes a norm, negative judgement of the credibility of spaced practices continues to prevail among many learners and educators, even though they might have somehow engaged in it through unconventional forms of learning, such as electronic technology, with or without realizing its advantages. However, this paper does not attempt to advocate that blocked practices should be replaced by pure spacing in science curricula, due to the nature of science learning and the complexity in the interplay between theory, practice, policy, and administration. Consequently, this paper suggests many collaborative research opportunities in a multitude of research areas, including cognitive and computer science, socio-political studies, and educational studies. The ultimate goal is not to speak against a certain traditional practice, but to promote a greater societal awareness of long-term learning growth and break down the obstacles to equitable science education for all students.

This paper is not without limitations. First, its scope is restricted in a theoretical account since it does not identify complexities in education policymaking and teacher training for spaced practice, as the spacing effect may not apply in every situation. Besides, the criteria for denoting cognitive processing characteristics unique for science education should be extended to different mental measurements beyond memory strength, since memory is also essential to other areas of education such as language, art, and humanities. Additional research findings should also explore the interplay between the spacing effect and interpretative learning skills, rather than the “memory consolidation” of interpretative skills. Finally, spaced learning may not always be suitable in every condition in science education, thereby lumping biology and mathematics together as science education that uses different sets of reasoning skills, while claiming the effectiveness of spacing effect underestimates the complexity of learning each subject in real life.
